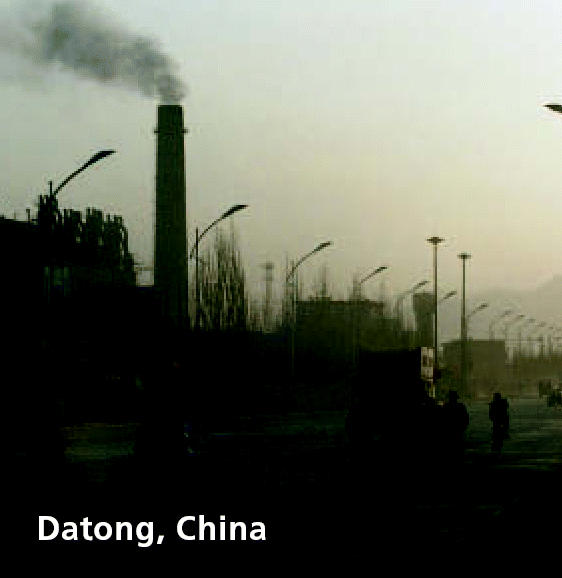# The Beat

**Published:** 2007-03

**Authors:** Erin E. Dooley

## Défense de Fumer

February 2007 saw something many believed could never happen: the banning of public smoking in France, a country often seen as staunchly pro-smoking. Public places as defined by the law include metro stations, museums, government offices, and stores, but not streets. Cafés, nightclubs, and restaurants have until January 2008 to comply with the ban. Individuals found lighting up will be fined about US$97, while the establishments where the person is found breaking the rules will be fined US$195. The French government will partially subsidize smoking cessation treatments to help residents quit smoking. In France, 60,000 deaths each year are directly linked to tobacco use, and 5,000 are attributed to secondhand smoke.

**Figure:**
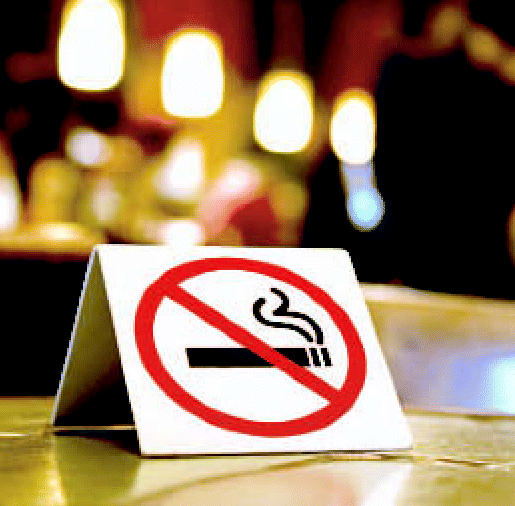


## Green Building Comes to DC

The District of Columbia passed legislation in December 2006 that makes it the first major city to require private developers to follow the Leadership in Energy and Environmental Design (LEED) standards of the U.S. Green Building Council. Under the law, district-funded commercial and housing projects beginning in 2008 must meet LEED standards. All commercial structures of 50,000 square feet or more must meet the standards by 2012. Separate standards for schools, still being developed by the council, are also to be adopted. Washington’s new baseball stadium is already being built in compliance with LEED standards.

## Will WIC Can Tuna?

A number of health advocacy groups have urged the USDA to remove canned tuna from its Special Supplemental Nutrition Program for Women, Infants, and Children, also known as WIC, saying the inclusion of tuna exposes breastfeeding mothers and their nursing infants to methylmercury when safer fish options exist. Though the agency plans to end an allowance for canned albacore tuna under the program, it may still offer light tuna, which critics say also can contain enough mercury to cause health effects. A 2005 Institute of Medicine review of the WIC program recommends offering canned salmon, which has far less mercury than tuna and costs only about 2¢ more per ounce. Over 250,000 women exclusively breastfeed as part of WIC, and canned tuna is offered as an incentive to those mothers who make this commitment. More than 8 million low-income mothers and their children receive WIC assistance each month. A final decision on tuna’s inclusion is expected in September 2007.

**Figure:**
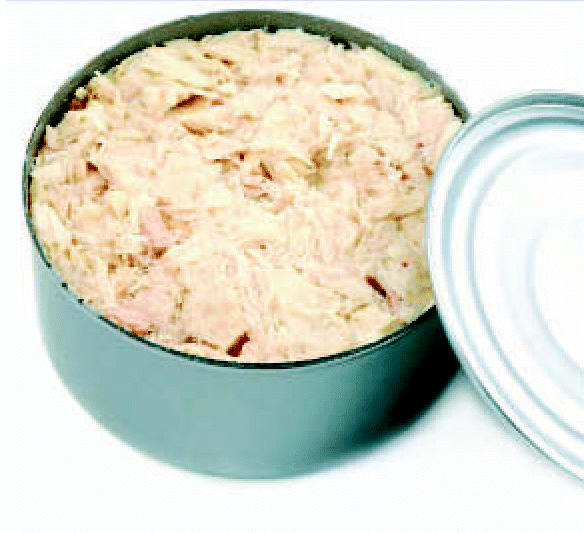


## A Friend Indeed

The Asthma and Allergy Foundation of America, in conjunction with the physician-led Allergy Standards Limited, has developed the first asthma friendly^®^ product standards for items such as plush toys, pillows and other bedding, flooring, vacuum cleaners, and air filtration devices. Products certified under the program are less likely to expose asthma and allergy sufferers to allergenic materials or chemical irritants. Certified items also come with instructions for keeping them “asthma friendly.” Stuffed animals, for instance, should be put in the freezer every four weeks and then washed to kill dust mites and their eggs. A list of certified products is available at http://www.asthmafriendly.com/.

**Figure:**
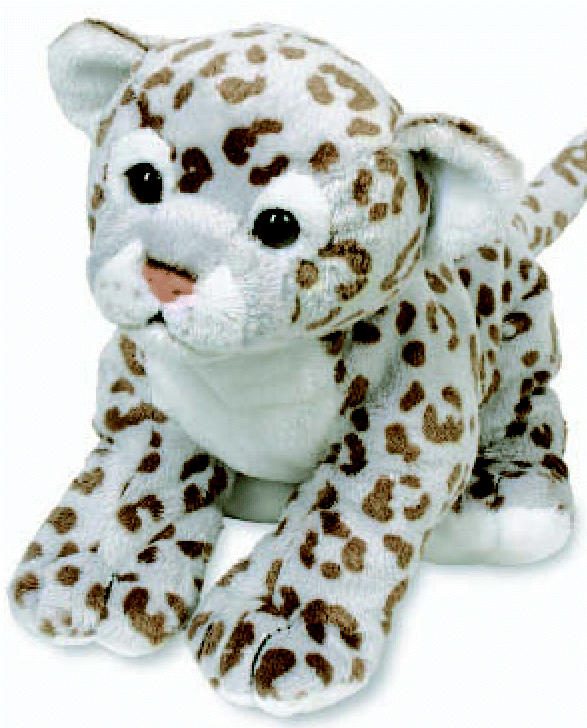


## Backyard Boiler Risk

In many areas of the United States, residents are using outdoor wood boilers to save money on heating oil and natural gas. These units are not equipped with air pollution controls, nor are they regulated, and owners are free to fuel them with anything that will burn, including painted wood and garbage. According to a study slated for the February 2007 issue of *Human and Ecological Risk Management*, the emissions from these units may significantly increase the risk of cancer, heart attack, and heart disease. People breathing the smoke from these boilers have a lifetime cancer risk of 1 in 1,000—practically the same odds faced by a cigarette smoker. According to estimates by the Michigan Department of Environmental Quality, in one hour of use a typical outdoor wood boiler may emit 160 g of toxics including benzene, dioxins, and polycyclic aromatic hydrocarbons.

## Young Lungs in China

As China’s economy booms, so does its air pollution. This, according to a November 2006 Chinese health report, is a main reason why increasing numbers of Chinese people in their 30s are now beset with chronic lung diseases that traditionally have affected mostly elderly people. The report says about 43 million people in China are affected by chronic lung diseases such as emphysema and chronic bronchitis, with about 1 million of these dying each year. Smoking was also named as a culprit in the rise of these diseases.

**Figure:**